# The genome sequence of a carabid beetle, *Carabus problematicus *Herbst, 1786

**DOI:** 10.12688/wellcomeopenres.23689.1

**Published:** 2025-02-10

**Authors:** Duncan Sivell, Olga Sivell, Ryan Mitchell, Natural History Museum Genome Acquisition Lab, Darwin Tree of Life Barcoding collective, Wellcome Sanger Institute Tree of Life Management, Samples and Laboratory team, Wellcome Sanger Institute Scientific Operations: Sequencing Operations, Wellcome Sanger Institute Tree of Life Core Informatics team, Tree of Life Core Informatics collective, Darwin Tree of Life Consortium

**Affiliations:** 1 Natural History Museum, London, England, UK; 2 Independent researcher, Sligo, County Sligo, Ireland

**Keywords:** Carabus problematicus, carabid beetle, genome sequence, chromosomal, Coleoptera

## Abstract

We present a genome assembly from an individual female specimen of *Carabus problematicus* (carabid beetle; Arthropoda; Insecta; Coleoptera; Carabidae). The genome sequence has a total length of 254.00 megabases. Most of the assembly (97.1%) is scaffolded into 14 chromosomal pseudomolecules, including the X sex chromosome. The mitochondrial genome has also been assembled and is 21.42 kilobases in length. Gene annotation of this assembly on Ensembl identified 12,311 protein-coding genes.

## Species taxonomy

Eukaryota; Opisthokonta; Metazoa; Eumetazoa; Bilateria; Protostomia; Ecdysozoa; Panarthropoda; Arthropoda; Mandibulata; Pancrustacea; Hexapoda; Insecta; Dicondylia; Pterygota; Neoptera; Endopterygota; Coleoptera; Adephaga; Caraboidea; Carabidae; Carabinae; Carabini; Carabina; *Carabus*; *Mesocarabus*; *Carabus problematicus* Herbst, 1786 (NCBI:txid49291)

## Background

*Carabus* ( *Mesocarabus*) *problematicus* Herbst, 1786 (Coleoptera, Carabidae) is a European species with a boreo-temperate distribution that extends from the Pyrenees and Alps north to the Faroes, Shetland and Finnmark, with few records east of Austria and Germany ( Anderson *et al.*, 2000; GBIF Secretariat, 2023).

*Carabus problematicus* is a common British species and similar in appearance to *C. violaceus*. These are large black ground beetles (20–30 mm long) with a distinctive purple or violet border around the edges of the pronotum and elytra. These species can usually be separated by their elytra, which tend to be obviously sculptured in *C. problematicus* but almost smooth in *C. violaceus.* Additional characters should be checked, however, as the texture of the elytra can vary in both species. The hind angles of the pronotum, which are raised higher in *C. problematicus* than in *C. violaceus*, are often used to confirm identifications ( Duff, 2012; James, 2018; Lindroth, 1974; Luff, 2007; Walters, 2012). Luff (1993) provides a key to the larvae of British *Carabus* species.

Both of these beetles are widespread in Britain and have overlapping distributions, but *C. problematicus* is more common further north and on higher ground while *C. violaceus* is commoner in the south and at lower elevations ( Duff, 2018; Lazenby, 2011; Luff, 1998). Only *C. problematicus* is present in Ireland ( Anderson *et al.*, 2000; Luff, 1998). There is some overlap in the habitat preferences of these species and they can occur together, but *C. problematicus* is more likely to be found in open environments such as heathland, moorland, rough grassland and open woodland, whereas *C. violaceus* is more typical of closed woodland, hedgerows, scrub, meadows, parks and gardens ( Brock, 2021; Duff, 2012; James, 2018; Lazenby, 2011; Lindroth, 1974; Lindroth, 1992; Luff, 1998; Luff, 2007).

*Carabus problematicus* adults and larvae are nocturnal and generalist predators of other invertebrates such as slugs, snails, earthworms and insect larvae ( Hammond *et al.*, 2019; Lindroth, 1985; Lindroth, 1992). This diet might be supplemented with plant buds and berries ( Brown, 2013). The life cycle can be annual or biennial; some adults may live longer than two years and individuals might overwinter as an adult or a larva ( Butterfield, 1986; Lindroth, 1985; Luff, 1998). Larvae can be active during the winter in temperatures above 3 or 4 °C ( Betz, 1992; Luff, 2005). The biennial life cycle is especially prevalent in upland areas where females tend to lay eggs in their second year ( Butterfield, 1986). This mix of life-cycles means that *C. problematicus* adults can be recorded in any month of the year ( Duff, 2018).

Due to their similarity *C. problematicus* and *C. violaceus* have both been referred to as Violet Ground Beetles ( Brown, 2013; Luff, 1998), although this common name is sometimes reserved for *C. violaceus* ( James, 2018; Walters, 2012). The names Ridged Violet Ground Beetle ( Brock, 2021; Walters, 2012) and Rough Violet Ground Beetle have been used more specifically for *C. problematicus*.

The Irish and mainland British populations of *C. problematicus* are the subspecies *harcyniae* Sturm, 1815 ( Duff, 2012; Luff, 2007), which may be referred to as *gallicus* Géhin, 1885 in earlier literature ( Anderson *et al*., 2000; Luff, 1998). The subspecies *feroensis* Lapouge, 1910 occurs on the Outer Hebrides and Northern Isles ( Duff, 2012).

**Figure 1.**
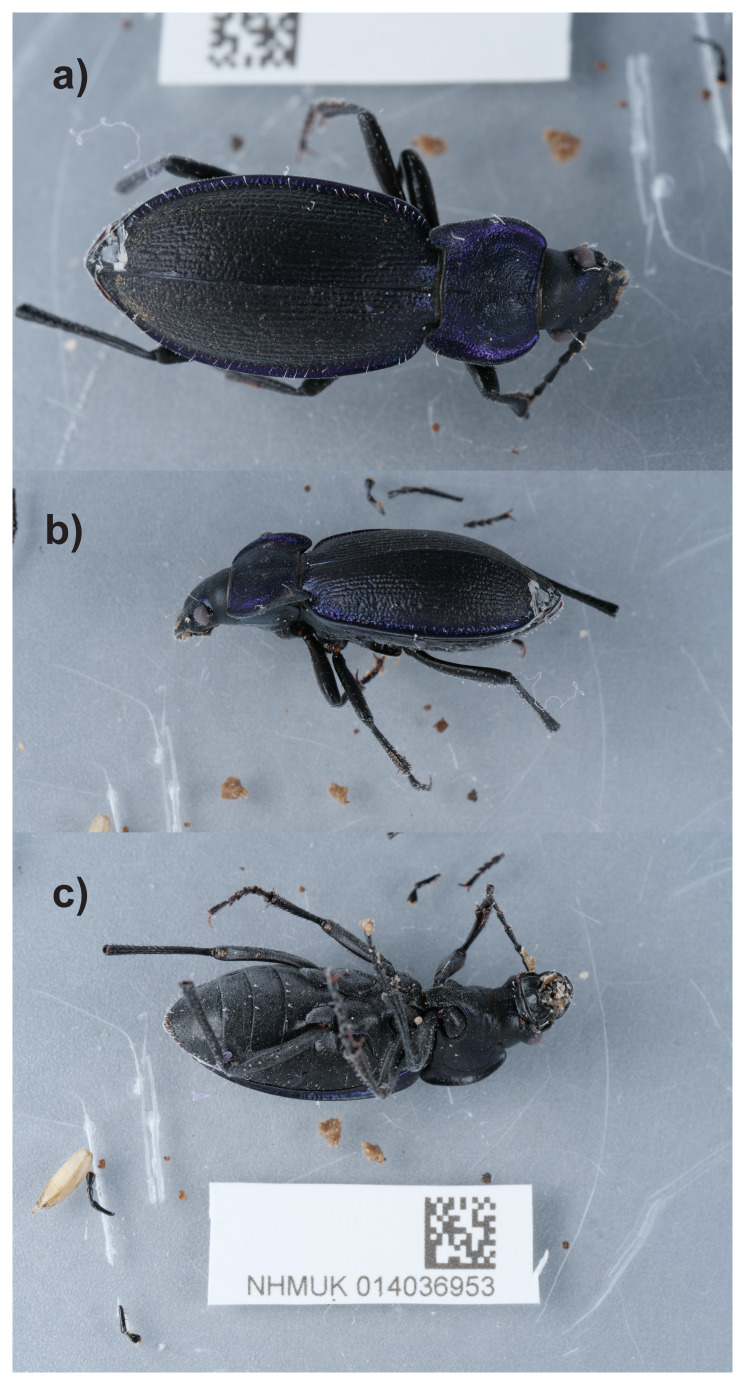
Photograph of the *Carabus problematicus* (icCarProb1) specimen used for genome sequencing.

Desender *et al*. (2005) have previously studied the population genetics of *C. problematicus* in Belgian woodlands. The genome of *Carabus problematicus* was sequenced as part of the Darwin Tree of Life Project, a collaborative effort to sequence all named eukaryotic species in the Atlantic Archipelago of Britain and Ireland. Here we present a chromosomally complete genome sequence for *Carabus problematicus*, based on a female specimen from Cothill Fen National Nature Reserve, England, United Kingdom ( Figure 1).

## Genome sequence report

The genome of *Carabus problematicus* ( Figure 1) was sequenced using Pacific Biosciences single-molecule HiFi long reads, generating a total of 27.71 Gb (gigabases) from 2.79 million reads, providing an estimated 111-fold coverage. Primary assembly contigs were scaffolded with chromosome conformation Hi-C data, which produced 95.33 Gb from 631.32 million reads. Specimen and sequencing details are summarised in Table 1.

**Table 1.** Specimen and sequencing data for *Carabus problematicus*.

Project information			
**Study title**	Carabus problematicus		
**Umbrella BioProject**	PRJEB61438		
**Species**	*Carabus problematicus*		
**BioSample**	SAMEA112221835		
**NCBI taxonomy ID**	49291		
Specimen information			
**Technology**	**ToLID**	**BioSample accession**	**Organism part**
**PacBio long read sequencing**	icCarProb1	SAMEA112222076	thorax
**Hi-C sequencing**	icCarProb1	SAMEA112222080	head
**RNA sequencing**	icCarProb1	SAMEA112222076	thorax
Sequencing information			
**Platform**	**Run accession**	**Read count**	**Base count (Gb)**
**Hi-C Illumina NovaSeq 6000**	ERR11271509	6.31e+08	95.33
**PacBio Sequel IIe**	ERR11263491	2.79e+06	27.71
**RNA Illumina NovaSeq 6000**	ERR11837487	5.95e+07	8.99
**RNA Illumina NovaSeq 6000**	ERR12035190	6.24e+07	9.42

Assembly errors, including 53 missing joins or mis-joins and 13 haplotypic duplications, were corrected by manual curation. This reduced the assembly length by 3.97% and the scaffold number by 6.3%, and increased the scaffold N50 by 13.19%. The final assembly has a total length of 254.00 Mb in 222 sequence scaffolds, with 85 gaps, and a scaffold N50 of 17.5 Mb ( Table 2).

**Table 2.** Genome assembly data for *Carabus problematicus*, icCarProb1.1.

Genome assembly		
Assembly name	icCarProb1.1	
Assembly accession	GCA_963422195.1	
*Accession of alternate haplotype*	*GCA_963422185.1*	
Span (Mb)	254.00	
Number of contigs	308	
Number of scaffolds	222	
Longest scaffold (Mb)	28.86	
Assembly metrics *		*Benchmark*
Contig N50 length (Mb)	4.3	*≥ 1 Mb*
Scaffold N50 length (Mb)	17.5	*= chromosome N50*
Consensus quality (QV)	56.2	*≥ 40*
*k*-mer completeness	Primary: 83.96%; alternate: 80.27%; combined: 99.38%	*≥ 95%*
BUSCO v5.4.3 lineage: endopterygota_odb10	C:99.0%[S:98.3%,D:0.7%], F:0.6%,M:0.4%,n:2,124	*S > 90%, D < 5%*
Percentage of assembly mapped to chromosomes	97.1%	*≥ 90%*
Sex chromosomes	X	*localised homologous pairs*
Organelles	Mitochondrial genome: 21.42 kb	*complete single alleles*
Genome annotation of assembly GCA_963422195.1 at Ensembl		
Number of protein-coding genes	12,311	
Number of non-coding genes	5,988	
Number of gene transcripts	26,283	

* Assembly metric benchmarks are adapted from Rhie *et al.* (2021) and the Earth BioGenome Project Report on Assembly Standards September 2024. ** BUSCO scores based on the endopterygota_odb10 BUSCO set using version 5.4.3. C = complete [S = single copy, D = duplicated], F = fragmented, M = missing, n = number of orthologues in comparison.

**Figure 2.**
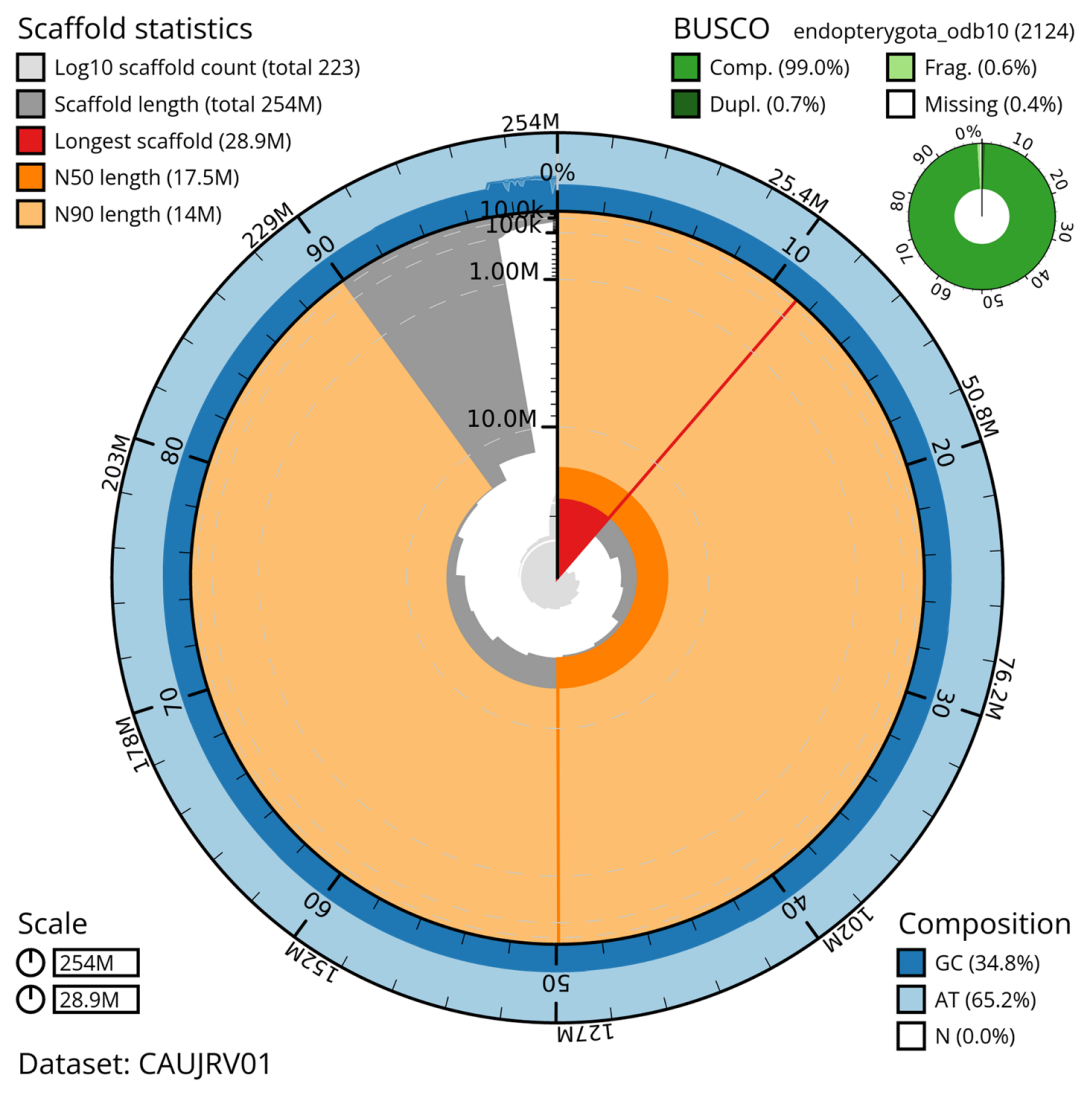
Genome assembly of *Carabus problematicus*, icCarProb1.1: metrics. The BlobToolKit snail plot provides an overview of assembly metrics and BUSCO gene completeness. The circumference represents the length of the whole genome sequence, and the main plot is divided into 1,000 bins around the circumference. The outermost blue tracks display the distribution of GC, AT, and N percentages across the bins. Scaffolds are arranged clockwise from longest to shortest and are depicted in dark grey. The longest scaffold is indicated by the red arc, and the deeper orange and pale orange arcs represent the N50 and N90 lengths. A light grey spiral at the centre shows the cumulative scaffold count on a logarithmic scale. A summary of complete, fragmented, duplicated, and missing BUSCO genes in the endopterygota_odb10 set is presented at the top right. An interactive version of this figure is available at https://blobtoolkit.genomehubs.org/view/CAUJRV01/dataset/CAUJRV01/snail.

**Figure 3.**
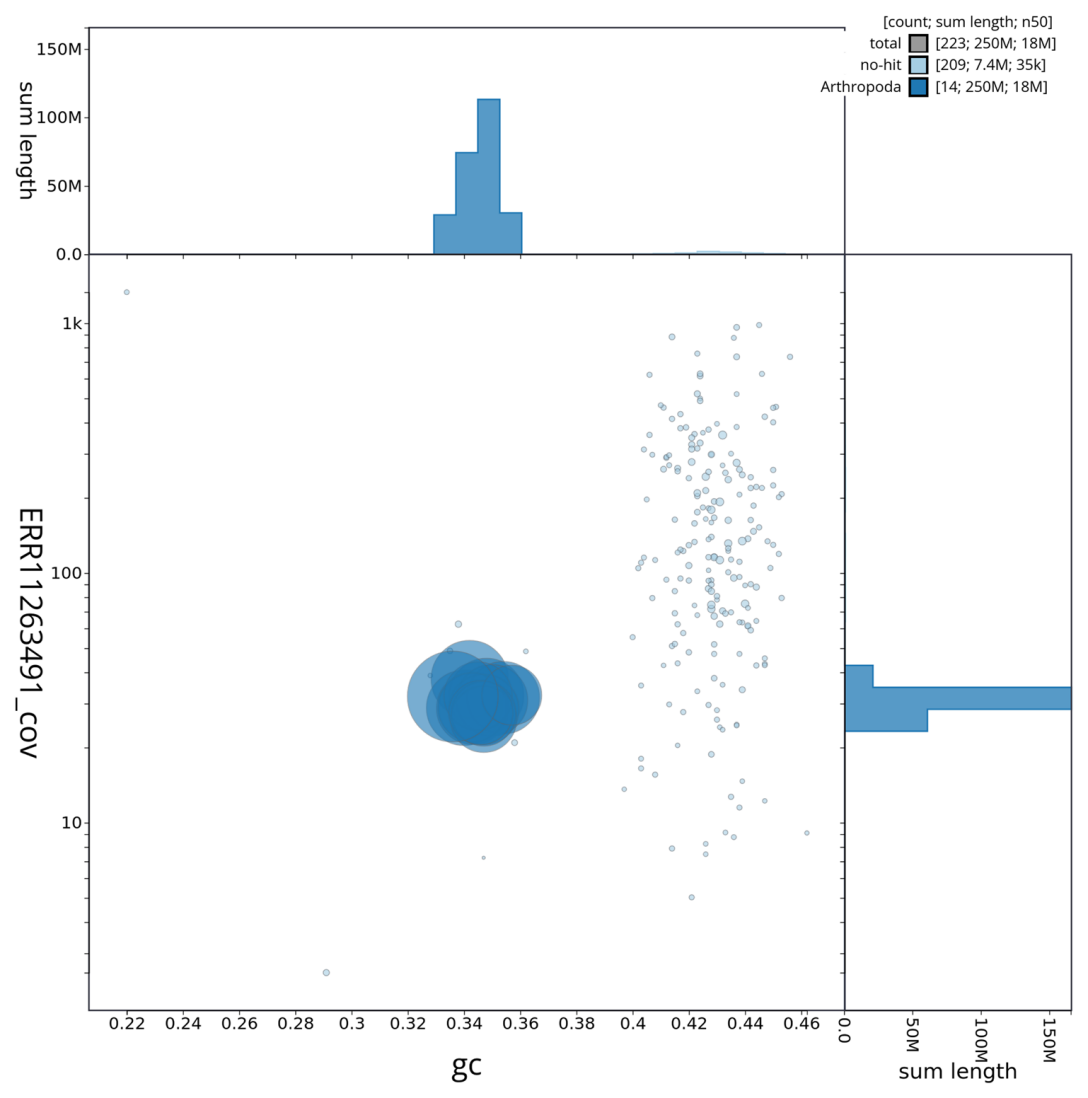
Genome assembly of *Carabus problematicus*, icCarProb1.1. BlobToolKit GC-coverage plot showing sequence coverage (vertical axis) and GC content (horizontal axis). The circles represent scaffolds, with the size proportional to scaffold length and the colour representing phylum membership. The histograms along the axes display the total length of sequences distributed across different levels of coverage and GC content. An interactive version of this figure is available at https://blobtoolkit.genomehubs.org/view/CAUJRV01/dataset/CAUJRV01/blob.

**Figure 4.**
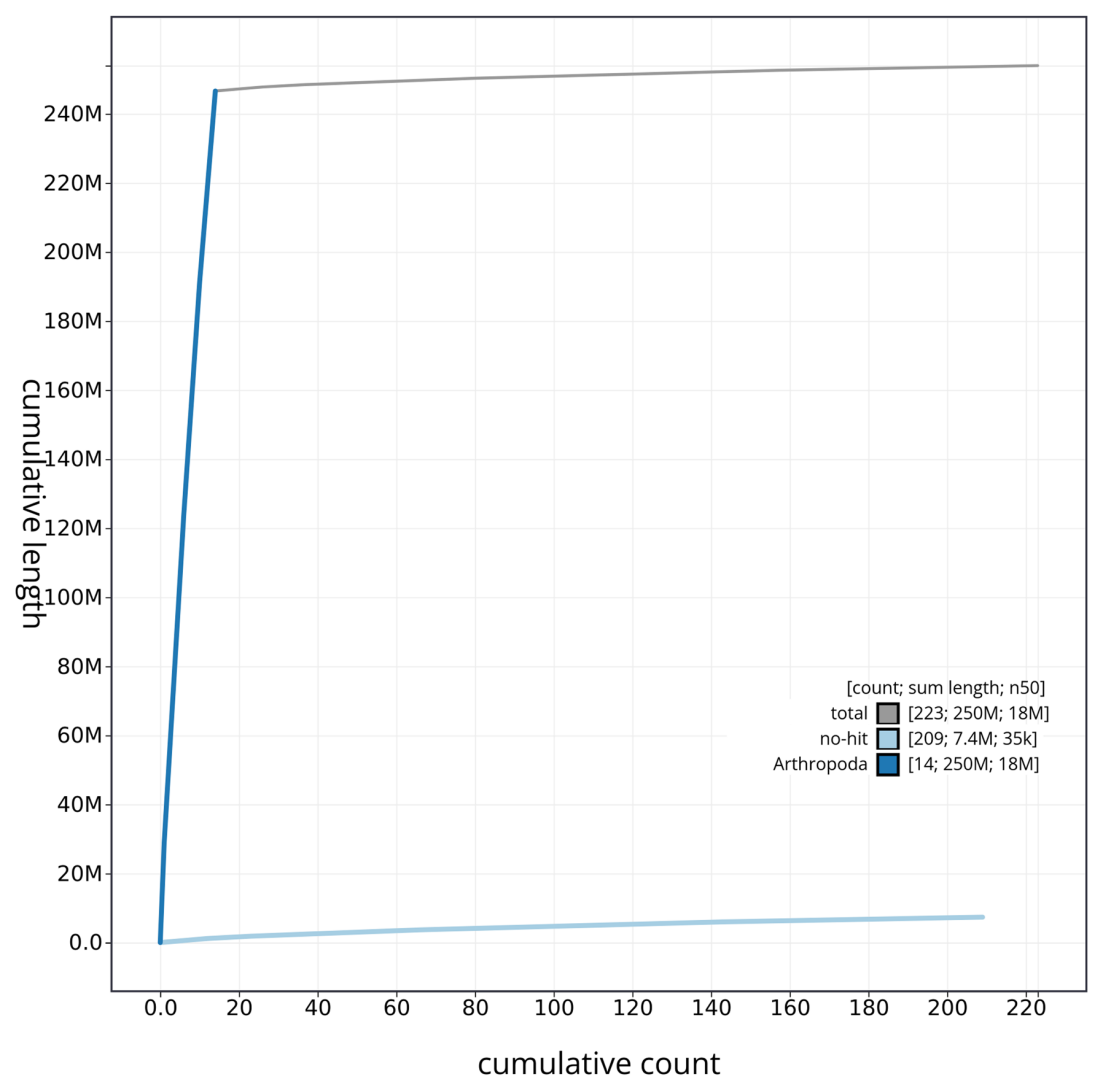
Genome assembly of *Carabus problematicus* icCarProb1.1: BlobToolKit cumulative sequence plot. The grey line shows cumulative length for all sequences. Coloured lines show cumulative lengths of sequences assigned to each phylum using the buscogenes taxrule. An interactive version of this figure is available at https://blobtoolkit.genomehubs.org/view/CAUJRV01/dataset/CAUJRV01/cumulative.

The snail plot in Figure 2 provides a summary of the assembly statistics, indicating the distribution of scaffold lengths and other assembly metrics. Figure 3 shows the distribution of scaffolds by GC proportion and coverage. Figure 4 presents a cumulative assembly plot, with separate curves representing different scaffold subsets assigned to various phyla, illustrating the completeness of the assembly.

**Figure 5.**
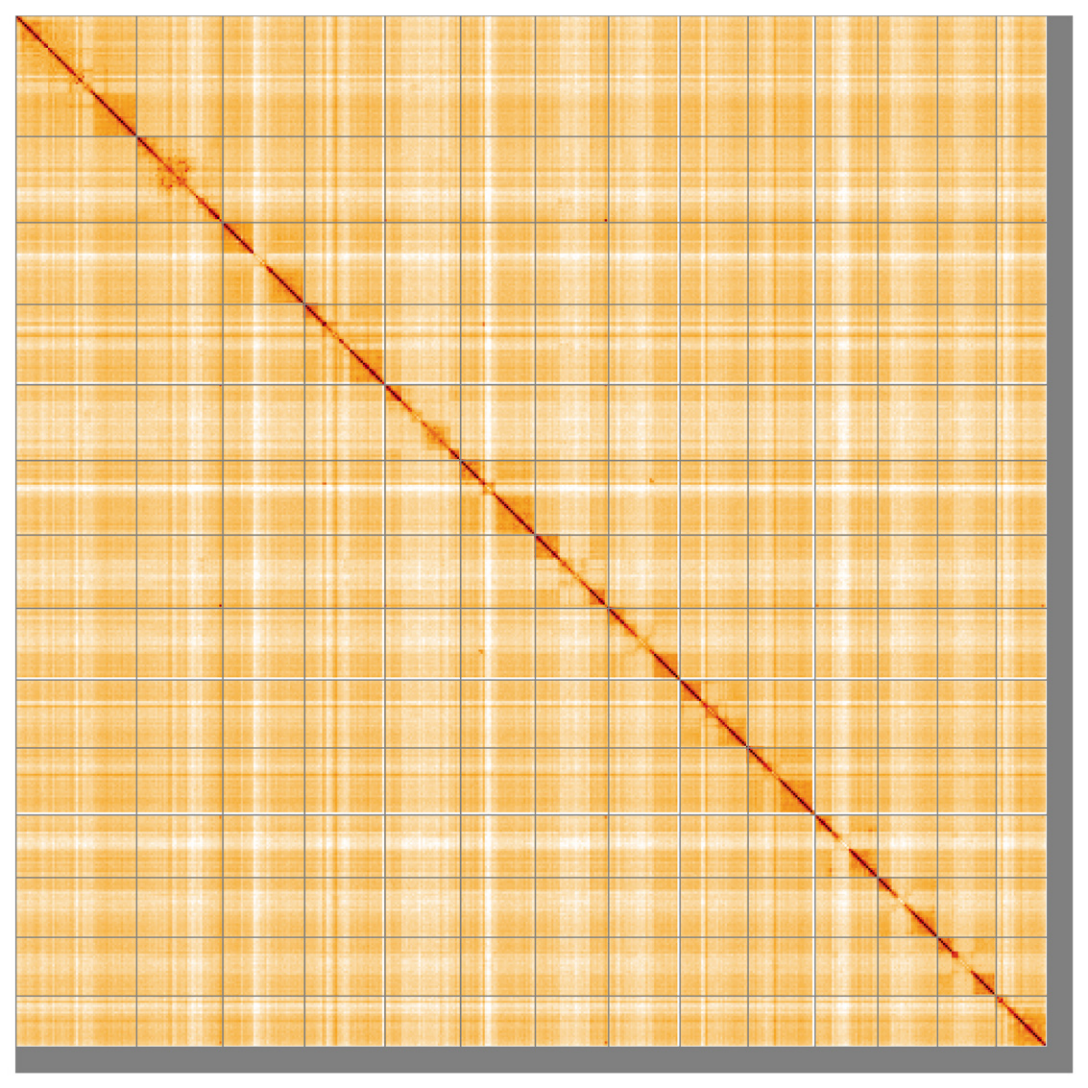
Genome assembly of *Carabus problematicus* icCarProb1.1: Hi-C contact map of the icCarProb1.1 assembly, visualised using HiGlass. Chromosomes are shown in order of size from left to right and top to bottom. An interactive version of this figure may be viewed at https://genome-note-higlass.tol.sanger.ac.uk/l/?d=SvNU1KlzT_q6OgSIDndgOA.

Most of the assembly sequence (97.1%) was assigned to 14 chromosomal-level scaffolds. These chromosome-level scaffolds, confirmed by the Hi-C data, are named in order of size ( Figure 5; Table 3). During manual curation, chromosome X was assigned based on synteny to the genome assembly of *Pterostichus niger* (GCA_947425015.1) ( Barclay *et al.*, 2023).

**Table 3.** Chromosomal pseudomolecules in the genome assembly of *Carabus problematicus*, icCarProb1.

INSDC accession	Name	Length (Mb)	GC%
OY728578.1	1	20.6	34.0
OY728579.1	2	19.58	34.0
OY728580.1	3	19.21	35.0
OY728581.1	4	18.12	35.5
OY728582.1	5	17.82	34.5
OY728583.1	6	17.52	35.0
OY728584.1	7	17.1	34.5
OY728585.1	8	16.28	34.5
OY728586.1	9	16.02	34.5
OY728587.1	10	14.99	34.5
OY728588.1	11	14.27	34.5
OY728589.1	12	14.05	34.5
OY728590.1	13	12.2	35.5
OY728577.1	X	28.86	33.5
OY728591.1	MT	0.02	22.0

While not fully phased, the assembly deposited is of one haplotype. Contigs corresponding to the second haplotype have also been deposited. The mitochondrial genome was also assembled and can be found as a contig within the multifasta file of the genome submission, and as a separate fasta file with accession OY728591.1.

The primary assembly has a Quality Value (QV) of 56.2. The *k*-mer completeness value was estimated as 99.38% for the combined assemblies (primary 83.96%; alternate 80.27%). BUSCO (v5.4.3) analysis using the endopterygota_odb10 reference set ( *n* = 2,124) indicated a completeness score of 99.0% (single = 98.3%, duplicated = 0.7%).

## Genome annotation report

The *Carabus problematicus* genome assembly (GCA_963422195.1) was annotated at the European Bioinformatics Institute (EBI) on Ensembl Rapid Release. The resulting annotation includes 26,283 transcribed mRNAs from 12,311 protein-coding and 5,988 non-coding genes ( Table 2; https://rapid.ensembl.org/Carabus_problematicus_GCA_963422195.1/Info/Index). The average transcript length is 6,752.30, with 1.44 coding transcripts per gene and 5.41 exons per transcript.

## Methods

### Sample acquisition and DNA barcoding

A female adult specimen of *Carabus problematicus* (specimen ID NHMUK014036953, ToLID icCarProb1) was collected from Cothill Fen National Nature Reserve, England, United Kingdom (latitude 51.69, longitude –1.32) on 2021-07-25. The specimen was collected by Ryan Mitchell, Olga Sivell, Duncan Sivell (Natural History Museum), identified by Duncan Sivell and then preserved by dry freezing at –80 °C.

The initial identification was verified by an additional DNA barcoding process according to the framework developed by Twyford *et al*. (2024). A small sample was dissected from the specimens and stored in ethanol, while the remaining parts were shipped on dry ice to the Wellcome Sanger Institute (WSI). The tissue was lysed, the COI marker region was amplified by PCR, and amplicons were sequenced and compared to the BOLD database, confirming the species identification ( Crowley *et al.*, 2023). Following whole genome sequence generation, the relevant DNA barcode region was also used alongside the initial barcoding data for sample tracking at the WSI ( Twyford *et al.*, 2024). The standard operating procedures for Darwin Tree of Life barcoding have been deposited on protocols.io ( Beasley *et al.*, 2023).

### Nucleic acid extraction

The workflow for high molecular weight (HMW) DNA extraction at the Wellcome Sanger Institute (WSI) Tree of Life Core Laboratory includes a sequence of procedures: sample preparation and homogenisation, DNA extraction, fragmentation and purification. Detailed protocols are available on protocols.io ( Denton *et al.*, 2023b). Tissue from the thorax was homogenised using a PowerMasher II tissue disruptor ( Denton *et al.*, 2023a).

HMW DNA was extracted in the WSI Scientific Operations core using the Automated MagAttract v2 protocol ( Oatley *et al.*, 2023). The DNA was sheared into an average fragment size of 12–20 kb in a Megaruptor 3 system ( Bates *et al.*, 2023). Sheared DNA was purified by solid-phase reversible immobilisation, using AMPure PB beads to eliminate shorter fragments and concentrate the DNA ( Strickland *et al.*, 2023). The concentration of the sheared and purified DNA was assessed using a Nanodrop spectrophotometer and Qubit Fluorometer using the Qubit dsDNA High Sensitivity Assay kit. Fragment size distribution was evaluated by running the sample on the FemtoPulse system.

RNA was extracted from thorax tissue of icCarProb1 in the Tree of Life Laboratory at the WSI using the RNA Extraction: Automated MagMax™ *mir*Vana protocol ( do Amaral *et al.*, 2023). The RNA concentration was assessed using a Nanodrop spectrophotometer and a Qubit Fluorometer using the Qubit RNA Broad-Range Assay kit. Analysis of the integrity of the RNA was done using the Agilent RNA 6000 Pico Kit and Eukaryotic Total RNA assay.

### Hi-C sample preparation

Tissue from the head of the icCarProb1 sample was processed at the WSI Scientific Operations core, using the Arima-HiC v2 kit. Tissue (stored at –80 °C) was fixed, and the DNA crosslinked using a TC buffer with 22% formaldehyde. After crosslinking, the tissue was homogenised using the Diagnocine Power Masher-II and BioMasher-II tubes and pestles. Following the kit manufacturer's instructions, crosslinked DNA was digested using a restriction enzyme master mix. The 5’-overhangs were then filled in and labelled with biotinylated nucleotides and proximally ligated. An overnight incubation was carried out for enzymes to digest remaining proteins and for crosslinks to reverse. A clean up was performed with SPRIselect beads prior to library preparation.

### Library preparation and sequencing

***PacBio HiFi***

At a minimum, samples were required to have an average fragment size exceeding 8 kb and a total mass over 400 ng to proceed to the low input SMRTbell Prep Kit 3.0 protocol (Pacific Biosciences, California, USA), depending on genome size and sequencing depth required. Libraries were prepared using the SMRTbell Prep Kit 3.0 (Pacific Biosciences, California, USA) as per the manufacturer's instructions. The kit includes the reagents required for end repair/A-tailing, adapter ligation, post-ligation SMRTbell bead cleanup, and nuclease treatment. Following the manufacturer’s instructions, size selection and clean up was carried out using diluted AMPure PB beads (Pacific Biosciences, California, USA). DNA concentration was quantified using the Qubit Fluorometer v4.0 (Thermo Fisher Scientific) with Qubit 1X dsDNA HS assay kit and the final library fragment size analysis was carried out using the Agilent Femto Pulse Automated Pulsed Field CE Instrument (Agilent Technologies) and gDNA 55kb BAC analysis kit.

Samples were sequenced using the Sequel IIe system (Pacific Biosciences, California, USA). The concentration of the library loaded onto the Sequel IIe was in the range 40–135 pM. The SMRT link software, a PacBio web-based end-to-end workflow manager, was used to set-up and monitor the run, as well as perform primary and secondary analysis of the data upon completion.

***Hi-C***

For Hi-C library preparation, DNA was fragmented using the Covaris E220 sonicator (Covaris) and size selected using SPRISelect beads to 400 to 600 bp. The DNA was then enriched using the Arima-HiC v2 kit Enrichment beads. Using the NEBNext Ultra II DNA Library Prep Kit (New England Biolabs) for end repair, a-tailing, and adapter ligation. This uses a custom protocol which resembles the standard NEBNext Ultra II DNA Library Prep protocol but where library preparation occurs while DNA is bound to the Enrichment beads. For library amplification, 10 to 16 PCR cycles were required, determined by the sample biotinylation percentage. The Hi-C sequencing was performed using paired-end sequencing with a read length of 150 bp on an Illumina NovaSeq 6000 instrument.

***RNA***

Poly(A) RNA-Seq libraries were constructed using the NEB Ultra II RNA Library Prep kit, following the manufacturer’s instructions. RNA sequencing was performed on the Illumina NovaSeq 6000 instrument.

### Genome assembly, curation and evaluation

***Assembly***

The HiFi reads were assembled using Hifiasm ( Cheng *et al.*, 2021) with the --primary option. Haplotypic duplications were identified and removed using purge_dups ( Guan *et al.*, 2020). The Hi-C reads were mapped to the primary contigs using bwa-mem2 ( Vasimuddin *et al.*, 2019). The contigs were further scaffolded using the provided Hi-C data ( Rao *et al.*, 2014) in YaHS ( Zhou *et al.*, 2023) using the --break option for handling potential misassemblies. The scaffolded assemblies were evaluated using Gfastats ( Formenti *et al.*, 2022), BUSCO ( Manni *et al.*, 2021) and MERQURY.FK ( Rhie *et al.*, 2020).

The mitochondrial genome was assembled using MitoHiFi ( Uliano-Silva *et al.*, 2023), which runs MitoFinder ( Allio *et al.*, 2020) and uses these annotations to select the final mitochondrial contig and to ensure the general quality of the sequence.

***Assembly curation***

The assembly was decontaminated using the Assembly Screen for Cobionts and Contaminants (ASCC) pipeline (article in preparation). Manual curation was primarily conducted using PretextView ( Harry, 2022), with additional insights provided by JBrowse2 ( Diesh *et al.*, 2023) and HiGlass ( Kerpedjiev *et al.*, 2018). Scaffolds were visually inspected and corrected as described by Howe *et al*. (2021). Any identified contamination, missed joins, and mis-joins were corrected, and duplicate sequences were tagged and removed. The curation process is documented at https://gitlab.com/wtsi-grit/rapid-curation (article in preparation).

***Assembly quality assessment***

The Merqury.FK tool ( Rhie *et al.*, 2020), run in a Singularity container ( Kurtzer *et al.*, 2017), was used to evaluate *k*-mer completeness and assembly quality for the primary and alternate haplotypes using the *k*-mer databases ( *k* = 31) that were computed prior to genome assembly. The analysis outputs included assembly QV scores and completeness statistics.

A Hi-C contact map was produced for the final version of the assembly. The Hi-C reads were aligned using bwa-mem2 ( Vasimuddin *et al.*, 2019) and the alignment files were combined using SAMtools ( Danecek *et al.*, 2021). The Hi-C alignments were converted into a contact map using BEDTools ( Quinlan & Hall, 2010) and the Cooler tool suite ( Abdennur & Mirny, 2020). The contact map was visualised in HiGlass ( Kerpedjiev *et al.*, 2018).

The genome was also analysed within the BlobToolKit environment ( Challis *et al.*, 2020) and BUSCO scores ( Manni *et al.*, 2021) were calculated.

Table 4 contains a list of relevant software tool versions and sources.

**Table 4.** Software tools: versions and sources.

Software tool	Version	Source
BEDTools	2.30.0	https://github.com/arq5x/bedtools2
BLAST	2.14.0	ftp://ftp.ncbi.nlm.nih.gov/blast/executables/blast+/
BlobToolKit	4.3.7	https://github.com/blobtoolkit/blobtoolkit
BUSCO	5.4.3 and 5.5.0	https://gitlab.com/ezlab/busco
bwa-mem2	2.2.1	https://github.com/bwa-mem2/bwa-mem2
Cooler	0.8.11	https://github.com/open2c/cooler
DIAMOND	2.1.8	https://github.com/bbuchfink/diamond
fasta_windows	0.2.4	https://github.com/tolkit/fasta_windows
FastK	427104ea91c78c3b8b8b49f1a7d6bbeaa869ba1c	https://github.com/thegenemyers/FASTK
Gfastats	1.3.6	https://github.com/vgl-hub/gfastats
GoaT CLI	0.2.5	https://github.com/genomehubs/goat-cli
Hifiasm	0.16.1-r375	https://github.com/chhylp123/hifiasm
HiGlass	44086069ee7d4d3f6f3f0012569789ec138f42b84a a44357826c0b6753eb28de	https://github.com/higlass/higlass
Merqury.FK	d00d98157618f4e8d1a9190026b19b471055b22e	https://github.com/thegenemyers/MERQURY.FK
MitoHiFi	2	https://github.com/marcelauliano/MitoHiFi
MultiQC	1.14, 1.17, and 1.18	https://github.com/MultiQC/MultiQC
NCBI Datasets	15.12.0	https://github.com/ncbi/datasets
Nextflow	23.04.0-5857	https://github.com/nextflow-io/nextflow
PretextView	0.2.5	https://github.com/sanger-tol/PretextView
purge_dups	1.2.3	https://github.com/dfguan/purge_dups
samtools	1.16.1, 1.17, and 1.18	https://github.com/samtools/samtools
sanger-tol/ascc	-	https://github.com/sanger-tol/ascc
Seqtk	1.3	https://github.com/lh3/seqtk
Singularity	3.9.0	https://github.com/sylabs/singularity
YaHS	version yahs-1.1.91eebc2	https://github.com/c-zhou/yahs

### Genome annotation

The Ensembl Genebuild annotation system ( Aken *et al.*, 2016) was used to generate annotation for the *Carabus problematicus* assembly (GCA_963422195.1) in Ensembl Rapid Release at the EBI. Annotation was created primarily through alignment of transcriptomic data to the genome, with gap filling via protein-to-genome alignments of a select set of proteins from UniProt ( UniProt Consortium, 2019).

### Wellcome Sanger Institute – Legal and Governance

The materials that have contributed to this genome note have been supplied by a Darwin Tree of Life Partner. The submission of materials by a Darwin Tree of Life Partner is subject to the **‘Darwin Tree of Life Project Sampling Code of Practice’**, which can be found in full on the Darwin Tree of Life website here. By agreeing with and signing up to the Sampling Code of Practice, the Darwin Tree of Life Partner agrees they will meet the legal and ethical requirements and standards set out within this document in respect of all samples acquired for, and supplied to, the Darwin Tree of Life Project. 

Further, the Wellcome Sanger Institute employs a process whereby due diligence is carried out proportionate to the nature of the materials themselves, and the circumstances under which they have been/are to be collected and provided for use. The purpose of this is to address and mitigate any potential legal and/or ethical implications of receipt and use of the materials as part of the research project, and to ensure that in doing so we align with best practice wherever possible. The overarching areas of consideration are:

•   Ethical review of provenance and sourcing of the material

•   Legality of collection, transfer and use (national and international)

Each transfer of samples is further undertaken according to a Research Collaboration Agreement or Material Transfer Agreement entered into by the Darwin Tree of Life Partner, Genome Research Limited (operating as the Wellcome Sanger Institute), and in some circumstances other Darwin Tree of Life collaborators.

## Data Availability

European Nucleotide Archive: Carabus problematicus. Accession number PRJEB61438; https://identifiers.org/ena.embl/PRJEB61438. The genome sequence is released openly for reuse. The *Carabus problematicus* genome sequencing initiative is part of the Darwin Tree of Life (DToL) project. All raw sequence data and the assembly have been deposited in INSDC databases. Raw data and assembly accession identifiers are reported in Table 1 and Table 2.

## Author information

Members of the Natural History Museum Genome Acquisition Lab are listed here: https://doi.org/10.5281/zenodo.12159242.

Members of the Darwin Tree of Life Barcoding collective are listed here: https://doi.org/10.5281/zenodo.12158331.

Members of the Wellcome Sanger Institute Tree of Life Management, Samples and Laboratory team are listed here: https://doi.org/10.5281/zenodo.12162482.

Members of Wellcome Sanger Institute Scientific Operations: Sequencing Operations are listed here: https://doi.org/10.5281/zenodo.12165051.

Members of the Wellcome Sanger Institute Tree of Life Core Informatics team are listed here: https://doi.org/10.5281/zenodo.12160324.

Members of the Tree of Life Core Informatics collective are listed here: https://doi.org/10.5281/zenodo.12205391.

Members of the Darwin Tree of Life Consortium are listed here: https://doi.org/10.5281/zenodo.4783558.
