# Supplementary material for: The genome sequence of a carabid beetle, Carabus problematicus Herbst, 1786
Source: Wellcome Open Res. 2025 Feb 10;10:59. [Version 1] doi: 10.12688/wellcomeopenres.23689.1 (PMC11880759; doi:10.12688/wellcomeopenres.23689.1)
Supplement: Peer review history

# Reviewer report: Reviewer response for version 1

**DOI:** 10.21956/wellcomeopenres.26135.r118970

**Published:** 2025-03-05

**Authors:** Marta Coronado-Zamora

**Affiliations:** 1 Institut Botanic de Barcelona, Barcelona, Catalonia, Spain

This data note describes the first high quality chromosomal assembly of the carabid beetle, *Carabus problematicus.* The genome data are available at NCBI BioProject, and data is accessible.  The genome assembly is highly contiguous, with overall very good assembly metrics. The mitochondrial genome is also included, as well as gene annotations.

- The RNA-seq was obtained only from thorax, and it was used to annotate genes with the Ensembl Rapid Release. Using only the transcriptome of this organismal part could represent a limitation in the completeness of the gene annotation?
- Remove labels a), b) and c) from Figure 1 if they are not used. 

Are sufficient details of methods and materials provided to allow replication by others?

Yes

Is the rationale for creating the dataset(s) clearly described?

Yes

Are the datasets clearly presented in a useable and accessible format?

Yes

Are the protocols appropriate and is the work technically sound?

Yes

Reviewer Expertise:

population genetics, transposable elements, genomics, adaptation

I confirm that I have read this submission and believe that I have an appropriate level of expertise to confirm that it is of an acceptable scientific standard.

# Reviewer report: Reviewer response for version 1

**DOI:** 10.21956/wellcomeopenres.26135.r119580

**Published:** 2025-03-04

**Authors:** Arun Arumugaperumal

**Affiliations:** 1 Department of Biotechnology, Rajalakshmi Engineering College, Chennai, Tamil Nadu, India

The data note presents the whole genome of the carabid beetle, *Carabus problematicus*. The genome assembly was done after long read sequencing and the final assembly was of size 254 Mb. The mitogenome was also assembled and protein-coding genes were predicted using Ensembl. BUSCO analysis showed 99% genome completeness.

 The author Ryan Mitchell can have an ORCID id. 

 Figure 1, a,b,c labels inserted but not mentioned in figure caption. The authors can take a call on retaining it if it is not used.

Are sufficient details of methods and materials provided to allow replication by others?

No

Is the rationale for creating the dataset(s) clearly described?

Yes

Are the datasets clearly presented in a useable and accessible format?

Yes

Are the protocols appropriate and is the work technically sound?

Yes

Reviewer Expertise:

Bioinformatics, Genomics

I confirm that I have read this submission and believe that I have an appropriate level of expertise to confirm that it is of an acceptable scientific standard.

# Reviewer report: Reviewer response for version 1

**DOI:** 10.21956/wellcomeopenres.26135.r119585

**Published:** 2025-03-01

**Authors:** Henrique Antoniolli

**Affiliations:** 1 Universidade Federal do Rio Grande do Sul (UFRGS), Porto Alegre, Brazil

In this article, the Sivell et al. present the first genome assembly of the carabid beetle *Carabus problematicus*. The assembly is highly contiguous, and most of the scaffolds have been assigned to pseudo-chromosomes. The authors have also identified the X chromosome based on synteny with another carabid species. The mitochondrial genome was also assembled and deposited along with the nuclear genome. The introduction section brings an overview about its biology, including key points about its morphology for a precise identification. The methods and protocols are clearly described, and all accession numbers for the data produced are properly indicated. The assembly metrics are satisfactory, therefore I recommend indexing the paper.

Are sufficient details of methods and materials provided to allow replication by others?

Yes

Is the rationale for creating the dataset(s) clearly described?

Yes

Are the datasets clearly presented in a useable and accessible format?

Yes

Are the protocols appropriate and is the work technically sound?

Yes

Reviewer Expertise:

Genomics, Evolution

I confirm that I have read this submission and believe that I have an appropriate level of expertise to confirm that it is of an acceptable scientific standard.
